# Phylogenetic evidence for the distinction of Saaremaa and Dobrava hantaviruses

**DOI:** 10.1186/1743-422X-2-90

**Published:** 2005-12-08

**Authors:** Tarja Sironen, Antti Vaheri, Alexander Plyusnin

**Affiliations:** 1 Department of Virology, Haartman Institute, Haartmaninkatu 3, FIN-00014 University of Helsinki, Finland

## Abstract

Dobrava virus (DOBV) and Saaremaa virus (SAAV) are two closely related hantaviruses carried by different rodent species. The distinction of these two viruses has been a matter of debate. While the phylogenies based on the viral M segment sequences were repeatedly showing monophyly of SAAV strains, some trees based on the S segment sequences were not, thus causing questions on the demarcation between these two viruses. In order to clarify this issue, the current collection of the virus S segment sequences was subjected to extensive phylogenetic analysis using maximum likelihood, maximum parsimony and distant matrix methods. In all inferred phylogenies, the SAAV sequences were monophyletic and separated from DOBV sequences, thus supporting the view that SAAV and DOBV are distinct hantavirus species. Since collection of the S segment sequences used in this study "obeyed" the molecular clock, calculations of the split of DOBV and SAAV were now repeated resulting in an estimation of 3.0–3.7 MYA that is very close to the values obtained earlier.

## Background

Hantaviruses (genus *Hantavirus*, family *Bunyaviridae*) are enveloped viruses with a segmented, single-stranded RNA genome of negative polarity [1]. The large (L) segment encodes the viral RNA polymerase, the medium (M) segment the two surface glycoproteins, and the small (S) segment the nucleocapsid protein (N). Hantaviruses cause two human zoonoses, hemorrhagic fever with renal syndrome (HFRS) in Eurasia and hantavirus pulmonary syndrome (HPS) in the Americas [reviewed in [2]]. DOBV is carried by yellow-necked mouse (*Apodemus flavicollis*) and is associated with severe HFRS in the Balkans (Slovenia, Albania and Greece). SAAV is carried by striped field mouse (*A. agrarius*) [3]. So far, the virus has been found in Estonia, the European part of Russia, Slovakia, Slovenia, Hungary, Denmark and Germany [2].

SAAV was initially called an *A. agrarius*-carried variant of Dobrava virus [3], but the accumulating data suggest that the virus should be regarded as a distinct hantavirus species. It is carried by a specific rodent host [3], there is a four-fold difference in two-way cross-neutralization tests [4], and the coexistence of SAAV and DOBV in the same geographic region [5,6] indicates reproductive isolation. They also exhibit 6.1–6.3% difference in the glycoprotein precursor amino acid sequences. This level is a fraction lower than the officially accepted 7% cut-off value [1]. It should be mentioned that some of the officially approved, distinct hantavirus species show lower than 7% diversity in their N or GnGc-sequences: Sin Nombre and New York viruses, Topografov and Khabarovsk viruses, Rio Mamore and Laguna Negra viruses, and Blood Land Lake and Prospect Hill viruses [7].

SAAV and DOBV also exhibit only 3% diversity on their N protein sequences. This unusually low level of diversity is most probably a reflection of host switching in their evolution [8,9]; this event seems to be historically recent (2.7–3.4 MYA) and these two viruses are still diverging [8]. There is another important feature differentiating DOBV and SAAV, and that is the apparently different pathogenicity in humans: while DOBV causes severe HFRS in humans, SAAV causes a milder form of the disease, similar to nephropathia epidemica [2]. This difference is also reflected in different pathogenicity in suckling mice: DOBV is lethal to suckling mice, while SAAV is not [10].

The phylogenetic distinction of SAAV and DOBV was recently a matter of debate [11,12]. While the phylogenies based on the M segment/GnGc protein sequences were repeatedly showing monophyly of SAAV strains, some trees based on the S segment/N protein sequences were not [[11,13], and our unpublished observations], thus causing questions on the demarcation between these two viruses. In order to clarify this issue, the current collection of DOBV and SAAV S segment sequences was subjected to extensive phylogenetic analysis. Especially important additions to the dataset include an *A. agrarius *-derived SAAV strain from Denmark, Saaremaa/Lolland/Aa1403/2000 [AJ616854), and two DOBV sequences from southern Russia, P-s1223/Krasnodar-2000 (AF442623) and As-1/Goryachiy Klyuch-2000 (AF442622). Our earlier data indicated that these sequences could be helpful for resolving the S phylogeny [14].

## Results and discussion

Our analysis was restricted to nt 37–1232 of the S segment available for all the strains. This part of the S segment includes almost complete coding region for the N protein. Accession numbers for the sequences are given in Table 1.

**Table 1:** Sequences used in the analysis

	Strain	Accession number
Saaremaa virus (SAAV)	Saaremaa/160 V	AJ009773
	90Aa/97	AJ009775
	Lolland/Aa1403/2000	AJ616854
	Kurkino/44Aa/98	AJ131672
	Kurkino/53Aa/98	AJ131673
	East Slovakia/856/Aa	AJ269549
	East Slovakia/862/Aa	AJ269550
Dobrava virus (DOBV)	Slovenia	L41916
	East Slovakia/400Af/98	AY168576
	Ano-Poroia/9Af/1999	AJ410615
	Ano-Poroia/13Af/99	AJ410619
	As-1/Goryachiy Klyuch-2000	AF442622
	P-s1223/Krasnodar-2000	AF442623
Seoul virus (SEOV)	Gou3	AB027522
	L99	AF288299
	Z37	AF187082
	SR11	M34881
Hantaan virus (HTNV)	Ah09	AF285264
	84Fli	AY017064
	76–118	M14626
	Lr1	AF288294
Andes virus (ANDV)	AH-1	AF324902
Topografov virus (TOPV)	Ls136V	AJ011646
Sin Nombre virus (SNV)	NM H10	L25784
El Moro Canyon virus (ELMCV)	RM-97	U11427
Puumala virus (PUUV)	Sotkamo	X61035
Tula virus (TULV)	Moravia/5302v/95	Z69991

**Table 2:** Bootstrap and puzzle support values for DOBV and SAAVclades in phylogenetic trees calculated using different methods.

method	outgroup	support for: DOBV	support for: SAAV
maximum likelihood	SEOV	100	70
maximum likelihood	collection*	100	49
maximum likelihood	no outgroup	100	100

maximum parsimony	SEOV	100	75
maximum parsimony	collection*	100	75

distance matrix: Neighbor-joining	SEOV	100	84
distance matrix: Neighbor-joining	collection*	100	91

distance matrix: Fitch-Margoliash	SEOV	79	58
distance matrix: Fitch-Margoliash	collection*	100	79
distance matrix: Fitch-Margoliash	no outgroup	100	99

TreePuzzle**	SEOV	99	87
TreePuzzle	collection*	99	75

*A collection of hantavirus sequences including SNV, ANDV, ELMCV, TULV, TOPV, PUUV, SEOV strains SR11 and Gou3, HTNV strains 76–118 and 84Fli **Tamura-Nei was used as the nucleotide (nt) substitution model in TreePuzzle, as suggested by Modeltest.

Since recombinant sequences might influence phylogenetic reconstructions (e.g. by "breaking" the molecular clock [15]), we wanted to check whether the sequences used in this study included any recombinants ones. A similarity plot (Stuart Ray's SIMPLOT2.5) was created in order to visualize the pattern of similarity between the DOBV and SAAV S segment nucleotide sequences, and phylogenetic trees were created on partial sequences, that were possibly of recombinant origin. Although we have found some indications on a recombinant origin of the strain Lolland (in particular, nt 200–460 were most similar to the Estonian SAAV strains, while other regions, especially nt 1150–1450, were more similar to SAAV strains from Russia and Slovakia), they were not unequivocal. For instance, the SIMPLOT data were not mirrowed by a mosaic-like pattern of the N protein sequence of Lolland strain. Moreover, the presence of this sequence did not "break" the molecular clock (see below). The Lolland sequence was, therefore, not excluded from our data set.

**Figure 1:**
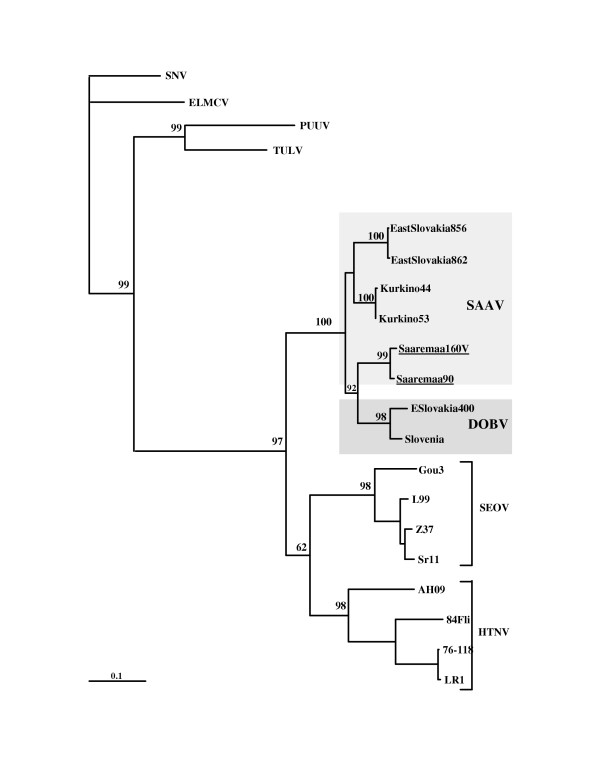
Phylogenetic tree created with TreePuzzle for a smaller data set. The tree is based on the nt 37–1232 of the S segment sequences.

**Figure 2:**
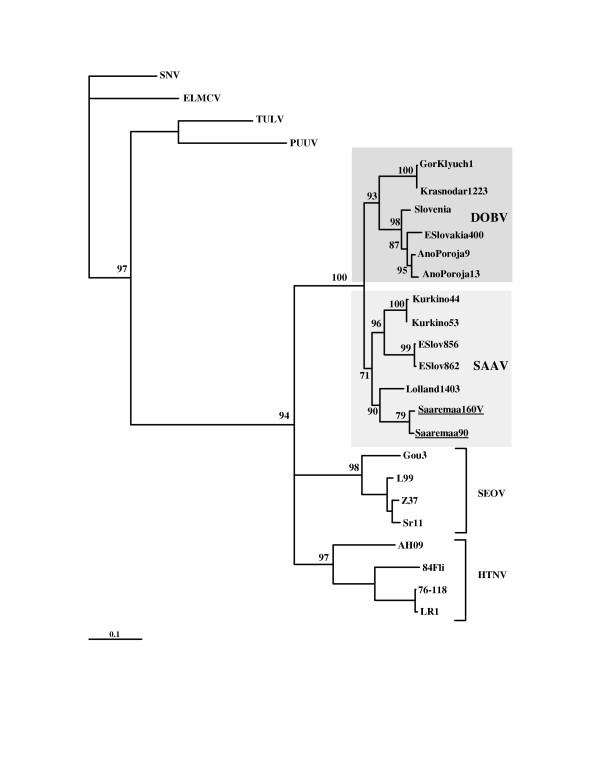
Phylogenetic tree created with TreePuzzle for a more representative data set. The tree is based on the nt 37–1232 of the S segment sequences. Two SAAV sequences that are placed differently on the trees shown on Fig. 1 and Fig 2 are underlined.

Next, we wanted to study whether the new additional sequences would have any effect on the clustering of DOBV and SAAV. A phylogenetic tree was re-calculated with the same collection of sequences and same parameters as has been done by Klempa et al. [11] (Fig. 1). The additional DOBV and SAAV sequences were then included to this set, a new phylogenetic tree was created, and indeed, a change in the topology was seen. The SAAV sequences turned monophyletic with a puzzle support of 71% (Fig. 2).

In order to confirm the phylogeny, trees were calculated using different algorithms listed earlier (Table 2). All methods agreed on placing DOBV and SAAV sequences into their own clusters. Placing of the two above mentioned DOBV sequences derived from southern Russia was more variable, but in most cases they were sharing a common ancestor with the other DOBV strains. The puzzle support values and bootstrap support for the DOBV cluster were in most cases very high (79–100%). For SAAV, the support was more variable, but only in two out of 12 phylogenies below the widely accepted confidentiality limit (70%) [16]. The support values were also varying depending on the phylogenetic algorithm, on the parameters used, and on the sequences chosen as outgroup. In the case of maximum likelihood trees, the use of additional hantavirus sequences as outgroup resulted in a lower bootstrap support for SAAV. In fact, a 100% support for SAAV monophyly was reached, when no outgroup sequences were used at all. This algorithm goes through an exhaustive search of all the possible trees, and it is possible that additional information creates an interfering noise to the phylogenetic signal. The opposite was happening with Fitch-Margoliash distance-matrix method. As more sequences were added, the bootstrap support for SAAV was increasing, most probably due to more accurate distance estimations. Nevertheless, in every tree, all the SAAV sequences were monophyletic and separated from DOBV. It should be stressed that bootstrap or puzzle support values do not estimate accuracy of a tree (i.e. right topology), but precision (how many trees had to be rejected) [17]. Phylogenies inferred here with different algorithms, and by varying the parameters used in the analyses (Table 2), gave a consensus answer on the monophyly of all SAAV strains, thus suggesting that this tree topology is most accurate.

Earlier it has been estimated, that the split of DOBV and SAAV happened 2,7–3.4 million years ago (MYA) (10). Since the larger collection of the S segment sequences used in this study "obeyed" the molecular clock, these calculations were now repeated resulting in an estimation of 3.0–3.7 MYA.

## Conclusion

In all phylogenies inferred in this study using different approaches such as maximum likelihood, maximum parsimony and distant matrices, the SAAV sequences were monophyletic and separated from DOBV sequences, thus supporting the view that SAAVand DOBV are distinct hantavirus species.

## Methods

Sequences were handled with BIOEDIT [18], and alignments were created using CLUSTALX [19]. The various methods used for phylogenetic analysis included maximum likelihood ("classic" maximum likelihood from PHYLIP [20] and TreePuzzle [21], maximum parsimony (PHYLIP) and distance matrix methods Neighbor joining and Fitch-Margoliash (PHYLIP). 500 boostrap replicates were used in PHYLIP programs and 10000 puzzling steps in TreePuzzle. MODELTEST and PAUP were used to check, which DNA substitution model would fit best to this data set [22,23]. The test for molecular clock and estimation of the time of split of these two viruses was done with TreePuzzle [21].

## Competing interests

The author(s) declare that they have no competing interests.

## Authors' contributions

TS carried out experiments, participated in the analysis of the results and drafted the manuscript. AV participated in the analysis of the results and helped to draft the manuscript. AP designed the study, participated in the analysis of the results and helped to draft the manuscript.
